# Ellagic acid on milk production performance, blood and milk hormones, antioxidant capacity and fecal microbial communities in lactating Yili mares

**DOI:** 10.3389/fmicb.2025.1656100

**Published:** 2025-08-13

**Authors:** Xinxin Huang, Linjiao He, Jun Ma, Yuqi Li, Jiahao Li, Changjiang Zang, Min Hou, Xiaobin Li

**Affiliations:** ^1^Xinjiang Key Laboratory of Herbivore Nutrition for Meat & Milk, College of Animal Science, Xinjiang Agricultural University, Ürümqi, China; ^2^Xinjiang Key Laboratory of Equine Breeding and Exercise Physiology, College of Animal Science, Xinjiang Agricultural University, Ürümqi, China; ^3^General Animal Husbandry Station of Ili Kazakh Autonomous Prefecture, Yining, China; ^4^General Animal Husbandry Station of Xinjiang Uygur Autonomous Region, Ürümqi, China; ^5^Xinjiang Laboratory of Special Environmental Microbiology, Institute of Applied Microbiology, Xinjiang Academy of Agricultural Sciences, Ürümqi, China

**Keywords:** ellagic acid, Yili mares, lactation performance, hormones, antioxidant, fecal microorganism

## Abstract

Ellagic acid (EA), a natural polyphenol, exerts potent antioxidant and anti-inflammatory effects in humans and other animals, while contributing to intestinal microbiota homeostasis. This study investigated the impact of EA supplementation on milk production, hormone secretion, antioxidant activity, and gut microbiota in lactating Yili mares. Eighteen lactating Yili mares with an average body weight of 400.06 ± 15.01 kg, average age of 9.89 ± 0.83 years, with similar parity (5–6 foalings) were used in this study. These mares had foaled in May (first foal born on May 7, last foal born on May 13) and had been lactating for 30 days at the initiation of the experiment. They were randomly allocated to 3 groups (n = 6 per group): a control group (CON) receiving no EA supplementation, the EA15 group (15 mg/kg BW/day EA), and the EA30 group (30 mg/kg BW/day EA). The supplementation trial commenced on lactation day 30 (study day 0) and continued for 90 days. By study days 60 and 90, EA supplementation enhanced milk production in lactating mares. On study day 30, serum prolactin (PRL) concentrations were increased in mares in the EA15 group, and milk PRL concentrations were increased in mares in the EA30 group compared to the CON group of mares. Conversely, serum luteinizing hormone (LH) concentrations and milk growth hormone (GH) concentrations were reduced. Compared to the CON group of mares, mares in the EA15 group had increased serum glutathione peroxidase (GSH-Px) activity, and mares in the EA30 group had increased milk superoxide dismutase (SOD) and catalase (CAT) activities, while reducing milk total antioxidant capacity (T-AOC) and malondialdehyde (MDA) levels. Supplementation with EA increased the relative abundance of Actinobacteriota, Verrucomicrobiota, Christensenellaceae, Coriobacteriales_Incertae_Sedis, *Christensenellaceae_R_7_group*, and *Phoenicibacter* in the feces of lactating mares, while decreasing the relative abundance of Proteobacteria, Moraxellaceae, and *Acinetobacter*. Overall, EA supplementation increases milk production in lactating Yili mares, modulates lactation-associated hormone secretion, improves the body’s antioxidant capacity, and alters the composition of the intestinal microflora. The results suggest potential applications of EA supplementation in equine nutrition strategies aimed at improving lactation performance and antioxidant status during lactation. Future research could focus on optimizing dosage regimens and validating its efficacy in larger-scale production systems to facilitate practical application in equine husbandry.

## Graphical abstract

The red upward arrow indicates an increase, and the blue downward arrow indicates a decrease.
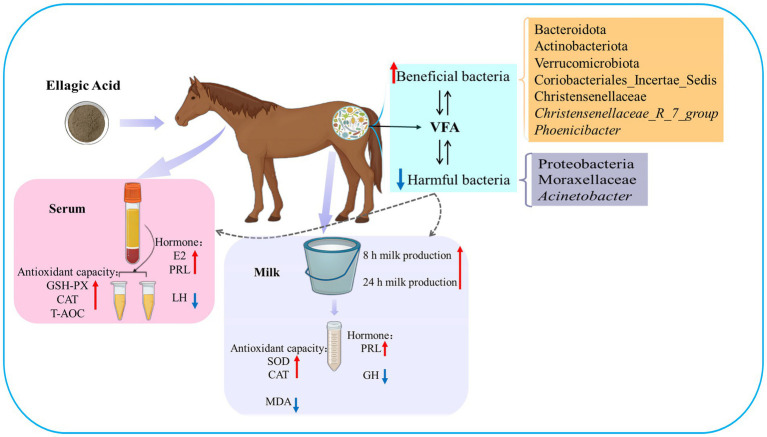

## 1 Introduction

Currently, horses are no longer limited to racing, tourism, and recreational purposes; their dairy products, valued for their unique nutritional composition and health benefits, are gaining increasing consumer acceptance (Rzekęć et al., 2020). Mare’s milk is highly nutritious, easily digestible, and closely resembles human milk in composition (Shaikh et al., 2022). Extensive research indicates that, compared to cow’s milk, prolonged consumption of mare’s milk and its fermented products (koumiss, chigee) serves as an ideal alternative for children with cow’s milk protein allergies and individuals with immunodeficiency or weakness health (Salimei and Fantuz, 2012). This is attributed to its high concentrations of whey protein, exogenous amino acids, lysozyme, and lactoferrin in mare’s milk, which confer notable health benefits (Musaev et al., 2021). Additionally, horse milk-based pharmaceuticals and cosmetics are widely utilized (Stuparu et al., 2016; Shaikh et al., 2022). As a result, the equine dairy industry is growing in many regions around the world, such as Western Europe (France, Belgium, The Netherlands), Central Asia (Kazakhstan, Kyrgyzstan), Russia, Germany, Italy, Greece, Mongolia, and China (Doreau and Martin-Rosset, 2011). However, mare’s milk production and quality are primarily influenced by diet, lactation stage, mammary gland health, and body health (Miraglia et al., 2020). During lactation, female livestock produce excessive oxygen free radicals as their energy demands and metabolic rates increase, which can easily lead to oxidative stress in the body (Berchieri-ronchi et al., 2011). In addition, the mammary glands of mares are small (<2 L) and cannot store large amounts of milk like the milk cisterns in cows, but have great lactation potential. Consequently, frequent udder stimulation is essential to maintain milk production during both non-milking and milking periods (Doreau and Martin-Rosset, 2002). During the non-milking phase (foals from birth to 1–2 months of age), foals nurse frequently to stimulate milk secretion, achieving successful suckling rates of 2–4 times per hour, lasting 55–75 s per session, with 40–72 suckling events per day (Cameron et al., 1999; Ninomiya et al., 2021). In the milking phase, under grazing conditions, mares are typically milked 4–6 times daily at 2–3 h intervals, excluding nighttime sessions. At peak lactation, some breeds undergo up to 8 daily milking sessions to maximize yield (Doreau and Martin-Rosset, 2011; Bat-Oyun et al., 2018). However, prolonged and frequent milking, coupled with increased metabolic demands during lactation, exacerbates oxidative stress and may predispose mares to subclinical mastitis (Canisso et al., 2021; Domańska et al., 2022). These physiological challenges not only compromise mare health but also impact milk production and quality.

Ellagic acid (EA), a naturally occurring plant-derived polyphenol, is widely distributed in fruits, vegetables, and nuts, existing in both free and as derivatives forms (Aishwarya et al., 2021). As a weak acid, EA has garnered significant interest due to its diverse biological activities, including anti-inflammatory, antibacterial, antioxidant, and antiviral properties, as well as its ability to modulate gut microbiota and enhance immune function (Rønning et al., 2020; Yang et al., 2022). Notably, EA interacts with intestinal microorganisms, not only altering microbial composition but also serving as a substrate for microbial metabolism, leading to the production of urolithins, which contribute to gut health (Zhang et al., 2023). In cattle, ruminal microorganisms play a critical role in maternal milk production and quality (Schären et al., 2018; Xue et al., 2018). Similarly, in non-ruminant herbivores, the hindgut (cecum and colon) functions as a fermentation chamber, harboring a complex microbial ecosystem that facilitates fiber digestion and nutrient utilization from forage (Harlow, 2015). Evidence suggests that the mechanisms governing milk synthesis in equines closely resemble those in ruminants, implying that alterations in hindgut microbiota may influence milk production and composition in mares (Doreau and Martin-Rosset, 2011). Given these insights, this study hypothesizes that under grazing conditions, EA supplementation in lactating Yili mares may leverage EA’s unique bioactivity and its regulatory effects on gut microbiota as a potential nutritional strategy to mitigate oxidative stress, enhance systemic health, increase milk production, and improve milk composition. Accordingly, this study aims to evaluate the impact of EA supplementation on milk production performance, milk composition, blood and milk hormone, antioxidant capacity, and fecal microbial diversity in lactating Yili mares under grazing conditions.

## 2 Materials and methods

This experiment was conducted in Zhaosu experimental Base of Xinjiang Agricultural University and was approved by the Animal Welfare and Ethics Review Committee of Xinjiang Agricultural University (Permission Number: 2022020). Which is complied with the Regulation of the Administration of Laboratory Animals (2017 Revision) issued by the State Council of China.

### 2.1 Sources of EA

EA was purchased from Wufeng Chicheng Biotechnology Co., Ltd. (Hubei, China). Light grey, powdery, its effective content ≥90%.

### 2.2 Animals and experimental design

The experiment was conducted from June to September 2022 at Kudeer Ranch (80.94°E, 42.94°N) in Zhaosu County, Ili Kazak Autonomous Prefecture, Xinjiang, China. A total of 18 lactating Yili mares with foals were selected from a larger cohort of 50 grazing mares. These mares, average body weight of 400.06 ± 15.01 kg (mean ± SD), average age of 9.89 ± 0.83 years (mean ± SD), with similar parity (5–6 foalings), had foaled in May (first foal born on May 7, last foal born on May 13) and had been lactating for 30 days at the start of the experiment. Throughout the trial, mares remained on continuous pasture grazing in mountain meadow grasslands until artificial milking ceased in October. The pasture vegetation primarily comprised *Carex liparocarpos*, *Festuca ovina* L., *Elymus sinkiangensis* D.F.Cui, *Phleum pratense* L., *Stipa capillata* L., Bromus inermis, *Poa annua* L., *Medicago falcata* L., *Phlomis umbrosa*, *Plantago asiatica* L., *Trifolium pratense* L., and various other forbs. Throughout the trial period, the pasture forage averaged 90.13% dry matter (air-dried basis), 10.17% crude protein, and 32.62% crude fiber. The nutritional levels of the air-dried forage consumed by lactating mares at different stages of the experiment are shown in Table 1. The 18 lactating Yili mares were randomly assigned to 3 groups (*n* = 6 per group): the control group (CON), which received no EA supplementation; the EA 15 group, supplemented with 15 mg/kg BW/day of EA; and the EA 30 group, supplemented with 30 mg/kg BW/day of EA. For the EA15 and EA30 groups, EA was precisely weighed daily using an electronic balance, then mixed with 50 g of bran and administered via individual muzzle feed bags. For the CON group, only 50 g of bran was placed in each mare’s individual muzzle feed bags. Supplementation was performed at 9 AM. The licking behavior of mares was monitored to ensure complete EA consumption. The supplementation dosages (15 mg/kg BW/day and 30 mg/kg BW/day) were based on findings by Li et al. (2023) in 1-year-old Thoroughbred horses. The trial commenced after 30 days of lactation (designated as day 0) and lasted 90 days.

**Table 1:** Nutrient levels of the air-dried forage at different stages of the experiment.

Nutrition levels^a^, % dry matter basis	Day 30 of the experiment	Day 60 of the experiment	Day 90 of the experiment
Dry matter	88.70	89.85	91.83
Organic matter	88.53	89.16	90.57
Crude ash	11.47	10.84	9.43
Ether extract	2.59	2.02	1.97
Crude fiber	24.68	37.99	35.20
Crude protein	10.69	10.05	9.76
Calcium	0.81	0.72	0.66
Phosphorus	0.14	0.14	0.15
Gross energy (MJ/kg)	17.43	17.74	16.36

a The nutritional levels were the measured values.

### 2.3 Feeding management

Mares and foals grazed together nightly in the Kudeer summer pasture, with unrestricted access to forage and water. Forage samples were collected using the cage-enclosure method to assess dietary nutrition (Xiao et al., 2020). Based on the foraging preferences of mares, five 2 m × 2 m cages were placed along their grazing routes in areas with uniform vegetation distribution. After 24 h of grazing, the vegetation within the cages was compared to the outside the cages to estimate and collect the forage consumed by the mares. Fresh forage inside the cages was clipped at ground level, and 500 g of forage was collected. It was placed in an oven at 65°C and dried to a constant weight for 48 h, then pulverized and ground through a 1 mm screen to determination of forage nutrient composition. Forage samples were collected at 30-day intervals throughout the experiment (days 30, 60, and 90). Lactating mares grazed on the summer pasture throughout the artificial milking period.

### 2.4 Sample collection and chemical analysis

#### 2.4.1 Feed analysis

Forage samples collected at each stage of the experimental period were analyzed for dry matter (DM) by drying in a ventilated oven at 105°C for 24 h. The ash content was determined by incineration in a muffle furnace at 550°C. The organic matter content was calculated using the equation: OM% = (100% − Ash%). The crude protein (*N* × 6.25) was determined via the Kjeldahl method (method 984.13; AOAC International, 2000), and crude fat was determined via ether extract (method 920.39; AOAC International, 2000). Crude fiber was determined using an Hanon 2000 automatic fiber analyzer (Hanon Group., Shandong, China) in accordance with Chinese national standards (Feed Industry, 2006). The contents of calcium and phosphorus were determined and analyzed by referring to the routine determination of nutritional components in Chinese national standards (Ministry of Science and Technology of the People’s Republic of China, 2001). Gross energy (GE) was determined and analyzed using an OR2014 high intelligent and high precision calorimeter (Shanghai Ourui Instrument Equipment Co., Ltd., Shanghai, China).

#### 2.4.2 Milk sampling and milk composition analysis

Milk production of each mare was collected and recorded on days 0, 30, 60, and 90 of the experiment. The physiological days of lactation in mares corresponding to the sampling time points during the experimental period are shown in Table 2. Under grazing conditions, mare milk production was estimated using the partial milking method. Given that milk secretion remains relatively stable over a 24-h cycle, daily milk production was inferred from measurements taken during a defined milking period. During the day, human hand-milking was conducted, while at night, foals remained with the mares for free grazing and suckling. Prior to each milking session, foals were allowed to suckle briefly to stimulate milk letdown, after which human hand-milking was performed. During hand-milking, mares and foals were physically separated, with foals tethered within visual range. Milk was fully extracted from the udders without oxytocin administration. Milking occurred every 2 h, 4 times daily (at 11:00, 13:00, 15:00, and 17:00). The yield from each session was recorded, and the cumulative yield from the four milkings represented the 8-h production estimate. Daily milk production was calculated using Saykin’s formula (Yao, 2011):

$${\text{Saykin}}^{’}s\;\text{formula}:W(\mathrm{kg}/d)=(G\times 24)/h$$

where W represents a day and night (24 h) milk production, G is the total yield from the actually measured period, 24 is number of hours in a day and night, and h is the isolation time between the mare and its foal during the milking period.

**Table 2:** Correspondence between experimental time points and lactation days in mares.

Experiment day	Lactation day
Day 0 of the experiment	Day 30 of lactation
Day 30 of the experiment	Day 60 of lactation
Day 60 of the experiment	Day 90 of lactation
Day 90 of the experiment	Day 120 of lactation

During the milking period, 50 mL milk samples were collected from each mare at each milking session and stored at 4°C. After the fourth milk sample collection of the day, the 4 milk samples were combined in equal proportions (1:1:1:1) and divided into 2 equal parts for further analysis.

One milk sample was immediately analyzed for milk composition: fat, protein, lactose, density, and solids-non-fat content using a portable milk quality analyzer (MILKOTESTER Master Eco, Henseki (Shanghai) Biotechnology Co., Ltd. Shanghai, China), and the measurements were repeated 3 times.

One milk sample was frozen at −20°C, and it was thawed completely and naturally at the laboratory room temperature. Milk concentration of estradiol (E2, H102-1-2), progesterone (PROG, H089-1-2), prolactin (PRL, H095-1-2), luteinizing hormone (LH, H206-1-2), and growth hormone (GH, H091-1-2) were measured using standard commercial ELISA kits (Nanjing Jiancheng Bioengineering Institute, Jiangsu, China) and microplate reader (Huadong Electronics DG5033A, Nanjing East China Electronics Group Medical Equipment Co., Jiangsu, China) following the manufacturer’s instructions. The milk contents of superoxide dismutase (SOD, A001-2-2), malondialdehyde (MDA, A0031-2), glutathione peroxidase (GSH-Px, A005-1-2), catalase (CAT, A007-1-1), total antioxidant capacity (T-AOC, A015-2-1) were measured using standard commercial kits (Nanjing Jiancheng Bioengineering Institute, Jiangsu, China) following the manufacturer’s instructions.

#### 2.4.3 Blood sampling and related index determination

On days 0 (i.e., mare lactation of 30 d), 30, 60, and 90 of the experimental period. Before the mare was supplemented with EA (i.e., at 08:30 AM), a blood sample was collected from the mare’s jugular vein by a veterinarian in 10 mL vacuum blood collection tubes without additives and centrifuged at 3,000 × g for 15 min at 4°C to obtain serum. The serum was aspirated into 2 mL Eppendorf tubes and stored appropriately at −80°C until further analyses.

The serum concentrations of E2, PROG, PRL, LH, GH, SOD, MDA, GSH-PX, CAT, and T-AOC were measured using the same methods as described above for milk samples.

#### 2.4.4 Fecal sampling and determination of pH and VFA

On day 30 of the experimental period, after blood sampling was completed and prior to EA supplementation of the mare, the mare was led to the horse restraint device and calmed by the breeder, and 500 g of feces were collected rectally by a veterinarian. After homogenizing, 5 g fecal samples were immediately taken and diluted in 15 mL of distilled water (1:3 feces to water) and stirred for 3–5 min at room temperature, the fecal pH was determined using a calibrated portable pH meter with an accuracy of 0.01 (FiveEasy22-Meter, Mettler-Toledo International Trading (Shanghai) Co., Shanghai, China), and each sample was measured 3 times and averaged. The remaining homogenized feces was divided into 3 subsamples (100 g) into sterile, enzyme-free freezing tubes, snap-frozen in liquid nitrogen, and stored at − 80°C until further analyses.

The concentrations of VFA (acetate, propionate, butyrate, isobutyrate, valerate, and isovalerate) in feces were determined by gas phase chromatography. Fecal subsamples were stored at −80°C, removed from −80°C, and followed overnight at 4°C until completely thawed, and homogenized. A fecal subsample (10 g) was mixed with 10 mL of ultrapure water, mixed and homogenized on a vortex meter for 30 min at 4°C, and filtered through four layers of gauze. The entire filtrate was centrifuged at 5,000 × g for 10 min, and the supernatant was placed at 4°C for 12 h. Transfer 0.5 mL of the supernatant to a 1.5 mL centrifuge tube, and 0.5 mL of 10% trichloroacetic acid (Sigma Aldrich (Shanghai) Trading Co. Shanghai, China) and 0.1 mL of 40 mmol of internal standard solutions (4-methylpentanoic acid, Sigma Aldrich (Shanghai) Trading Co. Shanghai, China) was added, mixed well using a vortex meter and allowed to stand for 20 min at 4°C. It was then centrifuged at 14,100 × g for 15 min and filtered through a 0.22 μm filter (Ranjeck Technology Co., Ltd. Beijing, China), and the filtrate (20 μL) was stored in a 2 mL glass vial (GC vials) used for analysis. The gas phase chromatography (Shimadzu GC2010, Shimadzu (China) Co., Ltd. Shanghai, China) was equipped with an Stabilwax (30 m × 0.25 mm (internal diameter) × 0.25 μm (film thickness)) column (Shimadzu, Shimadzu (China) Co., Ltd. Shanghai, China). The following parameters were set: the column oven temperature was increased from 55°C at 13°C/min to 200°C and held for 30 s. The inlet temperature was maintained at 230°C, the FID detector temperature at 240°C, and nitrogen was used as the carrier gas (flow rate 5.0 mL/min).

#### 2.4.5 DNA extraction from fecal samples, 16S rRNA sequencing, and bioinformatic analysis

A fecal subsample stored at −80°C was slowly thawed on ice and homogenized. Microbial genomic DNA was then extracted from the fecal sample using a Fecal Genomic DNA Extraction Kit (DP328, Tiangen Biotech (Beijing) Co., Ltd. Beijing, China). The concentration and purity of DNA were measured using a NanoDrop 2000 spectrophotometer, and the quality of DNA extraction was measured using 1% agarose gel electrophoresis. The hypervariable region V3–V4 of the bacterial 16S rRAN gene were amplified with barcode primers pairs 338F (5′-ACTCCTACGGGAGGCAGCA-3′) and 806R (5′-GGACTACHVGGGTWTCTAAT-3′). PCR products were recovered by 2% agarose gel, purified using the AxyPrep DNA Gel Extraction Kit (Axygen Biotechnology (Hangzhou) Limited. Hangzhou, China), eluted with Tris-HCl, detected by 2% agarose gel electrophoresis and quantified using Quanti Fluor-ST (Promega (Beijing) Biotech Co., Ltd. Beijing, China). The purified fragments were then used to construct libraries according to the standard procedure for the Illumina platform, and after the library was qualified, the Illumina NovaSeq 6000 was used for sequencing. Sequencing was performed by Beijing Biomarker Technologies Co., Ltd. (Beijing, China). The raw sequencing data has been submitted into the National Center for Biotechnology Information sequence read archive under PRJNA1123219.

After obtaining the raw sequencing data, Trimmomatic (version 0.33) (Bolger et al., 2014) was first used to filter the raw reads obtained from sequencing. Identification and removal of primer sequences was then performed using Cutadapt (version 1.9.1) (Martin, 2011) to obtain clean reads containing no primer sequences. The DADA2 (Callahan et al., 2016) method in QIIME2 (version 2020.6) was used for denoising, paired-end sequence splicing, and removal of chimeric sequences to obtain final non-chimeric reads. Sequences were clustered using USEARCH (version 10.0) (Edgar, 2013) based on 97% concordance, with a default threshold of 0.005% of all sequences to filter the operational taxonomic units (OTUs). SILVA was used as a reference database to annotate the feature sequences taxonomically using the Naive Bayes classifier to obtain the taxonomic information of the species corresponding to each feature, and then the community composition of the samples was counted at each level (phylum, class, order, family, genus, species). The QIIME software was then used to generate species abundance tables at different taxonomic levels, which were then plotted as community structure maps at each taxonomic level of the samples using R language tools (version 3.1.1). Alpha diversity indices were evaluated and calculated using QIIME2 (version 2020.6) software, including Chao1, Shannon, Simpson, ACE, Coverage and PD_whole_tree indices. Beta diversity analyses were performed using QIIME software to compare the degree of similarity in species diversity, including principal component analysis (PCA), principal coordinate analysis (PCoA), and nonmetric multidimensional scaling (NMDS) indices, between different samples. PCoA is evaluated at the OTUs level based on Weighted Unifrac distances. NMDS was calculated based on weighted UniFrac distances, and the differences in β-diversity between groups were assessed using permutational multivariate ANOVA (PERMANOVA) test. Linear discriminant analysis (LDA) coupled with effect size (LEfSe) analysis uses LEfSe software, and the default LDA score filter value is 4. The functions of microflora were predicted based on the Tax4Fun database.

### 2.5 Statistical analysis

All data analyses were performed using SAS version 8.1 (SAS Institute Inc., Cary, NC, United States). To evaluate the effect of the EA supplementation on milk yield, milk fat, milk protein, milk lactose, milk density, and solids-non-fat content in lactating mares, data were analyzed using the PROC MIXED procedure as follows:

$$Y_{\text{ijkl}}=\mu +E_{i}+T_{j}+B_{k}+ET_{\mathrm{ij}}+H_{l}+e_{\text{ijkl}}$$

where *Y_ijkl_* = the dependent variable; *μ* = is the overall mean; *E_i_* = fixed effect of EA supplementation levels; *T_j_* = the effect of sampling time [on days 0 (i.e., mare lactation of 30 d), 30, 60, and 90 of the experimental period]; *B_k_* = random block effect; *ET_ij_* = effect of the interaction between EA supplementation and sampling time; *H_l_* = the random effect of mare; *e_ijkl_* = residual error. The data on hormone concentration, antioxidative indices in serum and milk, and fecal pH and VFA that follow a normal distribution, they were analyzed using the LSD procedure, and selected based on the least Akaike information criterion value. The data are presented as LSM ± SEM, and *p* < 0.05 was considered significant difference. The correlation between indicators and fecal microorganisms was determined using Spearman’s correlation in the R package pheatmap (version 1.012) (Kolde, 2019). The histogram was generated using GraphPad Prism version 10.1.2 (GraphPad Software, San Diego, CA, United States).

## 3 Results

### 3.1 Milk production and composition in lactating mares

The main effects and interactions of EA supplementation on milk production and composition in lactating mares are shown in Table 3. On days 60 and 90, the milk production of mares in the EA15 group was higher than that in the CON group (*p* < 0.05), whereas no difference between the EA15 and EA30 groups. The treatment × time interaction had no significant effect on either the milk production or composition of the mares. On day 30, milk fat content of mares in the EA15 group was higher than that of mares in the CON group (*p* < 0.05). On day 60, milk density content of mares in the EA15 group was higher than that of mares in the EA30 group (*p* < 0.05). Throughout the experiment, milk production, protein, lactose, density, and solids-nonfat concentrations peaked on day 30 in all treatment groups and were higher than those on days 0, 60, and 90 of the experiment. However, fat content was higher on the 60th day of the trial than at other experimental time points.

**Table 3:** Effect of supplemental EA on milk production and composition in lactating mares.

Items	Treatments			SEM	*p*-value		
	CON	EA 15	EA 30		Treatments	Time	Treatments × time
8 h milk production, kg							
0 d	3.12^x^	3.13^x^	3.16^xy^	0.1296	0.0015	<0.0001	0.3881
30 d	3.16^x^	3.52^x^	3.56^x^				
60 d	2.65^by^	3.23^ax^	3.06^ay^				
90 d	1.72^bz^	2.09^ay^	1.89^abz^				
24 h milk production, kg							
0 d	9.36^x^	9.39^x^	9.48^xy^	0.3888	0.0015	<0.0001	0.3881
30 d	9.47^x^	10.56^x^	10.67^x^				
60 d	7.94^by^	9.70^ax^	9.19^ay^				
90 d	5.15^bz^	6.28^ay^	5.67^abz^				
Fat, %							
0 d	1.03^y^	1.47^xy^	1.35^y^	0.1409	0.0209	0.0002	0.4224
30 d	1.08^by^	1.53^axy^	1.47^aby^				
60 d	1.60^xy^	1.85^x^	1.90^x^				
90 d	1.43^x^	1.28^y^	1.43^y^				
Protein, %							
0 d	1.82^x^	1.88^x^	1.78^x^	0.0306	0.0315	<0.0001	0.4619
30 d	1.87^x^	1.83^x^	1.77^x^				
60 d	1.72^y^	1.75^y^	1.73^x^				
90 d	1.63^z^	1.67^z^	1.62^y^				
Lactose, %							
0 d	6.33^y^	6.43^xy^	6.33^x^	0.0586	0.0305	<0.0001	0.7364
30 d	6.47^x^	6.57^x^	6.33^x^				
60 d	6.27^y^	6.30^yz^	6.23^x^				
90 d	6.12^z^	6.13^z^	6.08^y^				
Density, kg/m^3^							
0 d	1032.95^x^	1033.38^x^	1032.72^x^	0.2792	0.0234	<0.0001	0.6326
30 d	1033.48^x^	1032.83^xy^	1032.60^x^				
60 d	1032.00^aby^	1032.07^ayz^	1031.62^by^				
90 d	1031.15^z^	1031.37^z^	1030.75^y^				
Solids-non-fat, %							
0 d	8.98^xy^	9.15^x^	9.02^x^	0.0803	0.2025	<0.0001	0.7502
30 d	9.13^x^	9.02^xy^	8.97^x^				
60 d	8.82^y^	8.85^y^	8.77^x^				
90 d	8.55^z^	8.58^z^	8.45^y^				

CON: the control group, receiving no EA supplementation (*n* = 6); EA 15: the EA 15 group, each mare was supplemented with EA 15 mg/kg BW every day (*n* = 6); EA 30: the EA 30 group, each mare was supplemented with EA 30 mg/kg BW every day (*n* = 6). SEM, standard error of the mean. Different letters in the same row indicate significant differences (^a,b^*p* < 0.05) Different letters in the same column indicate significant differences (^w–y^*p* < 0.05).

### 3.2 Serum and milk hormone concentrations in lactating mares

**Figure 1:**
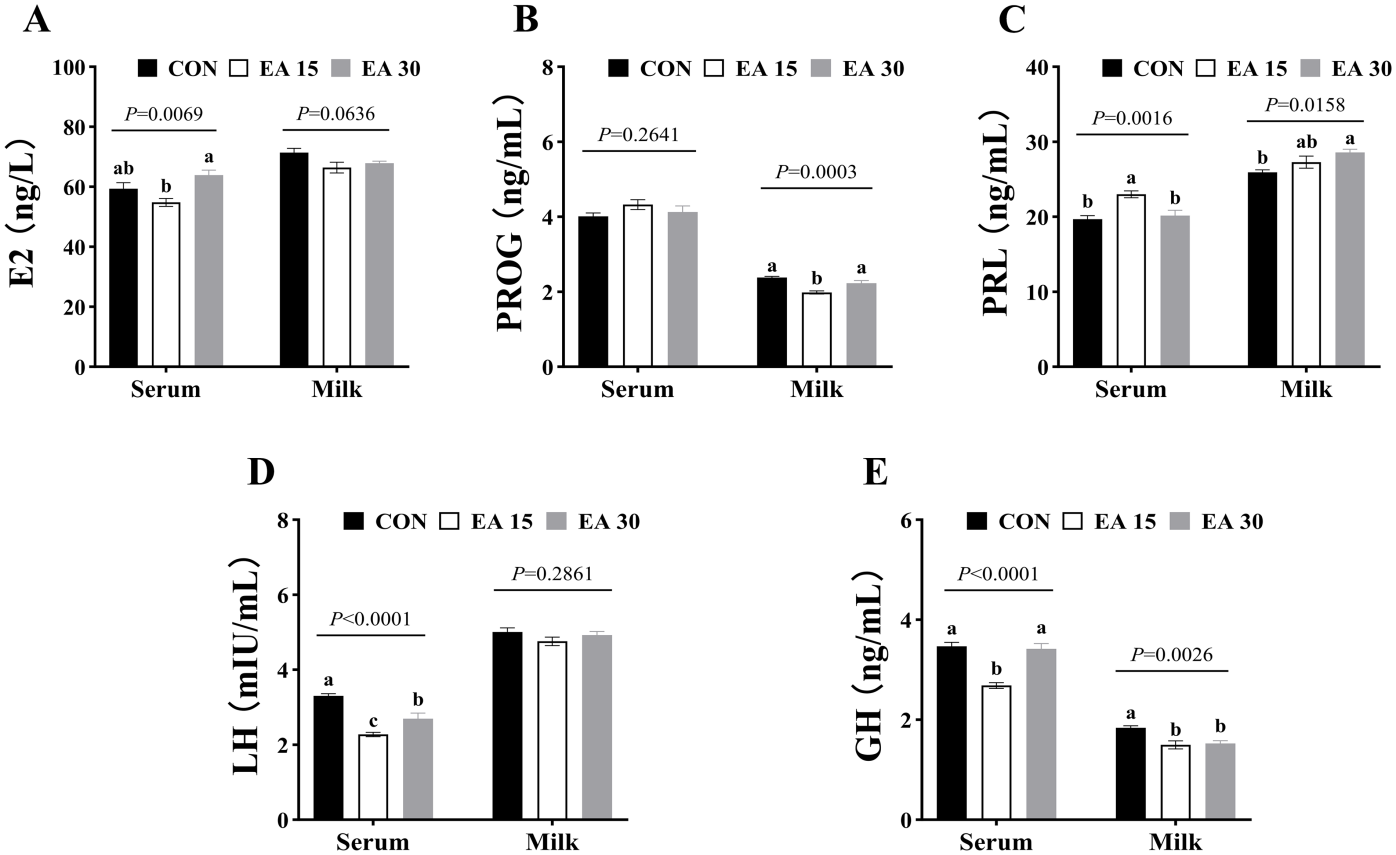
Effect of supplemental EA on serum and milk hormone concentrations in lactating mares on day 30 of the experiment. Effect of supplemental EA on serum and milk E2 concentrations in lactating mares **(A)**, serum and milk PROG concentrations **(B)**, serum and milk PRL concentrations **(C)**, serum and milk LH concentrations **(D)**, serum and milk GH concentrations **(E)**. Values are means with their standard errors represented by vertical bars. Different letters in the same bar chart represent significant differences (^a–c^*p* < 0.05). E2, estradiol; PROG, progesterone; PRL, prolactin; LH, luteinizing hormone; GH, growth hormone. CON: the control group, receiving no EA supplementation (*n* = 6); EA 15: the EA 15 group, each mare was supplemented with EA 15 mg/kg BW every day (*n* = 6); EA 30: the EA 30 group, each mare was supplemented with EA 30 mg/kg BW every day (*n* = 6).

The effects of EA supplementation on serum and milk hormone concentrations in lactating mares on day 30 of the trial are shown in Figure 1. Serum E2 concentrations of mares in the EA30 group was higher than that in the EA15 group (*p* = 0.0069; Figure 1A). Milk PROG concentrations of mares in the EA15 group were decreased compared to those in the CON and the EA30 groups (*p* = 0.0003; Figure 1B). Serum PRL concentrations of mares in the EA15 group was higher than that in the CON group and the EA30 groups (*p* = 0.0016), whereas milk PRL concentrations of mares in the EA30 group was higher than that in the CON group (*p* = 0.0158; Figure 1C). Serum LH concentrations were lower in mares in both the EA15 and EA30 groups compared to mares in the CON group, and mares in the EA15 group were lower than mares in the EA30 group (*p* < 0.0001; Figure 1D). Serum GH concentrations were lower in mares in the EA15 group than in mares in the CON and EA30 groups (*p* < 0.0001), while milk GH concentrations were reduced in both the EA15 and EA30 groups compared to the CON group (*p* = 0.0026; Figure 1E).

### 3.3 Antioxidative status in serum and milk of lactating mares

**Figure 2:**
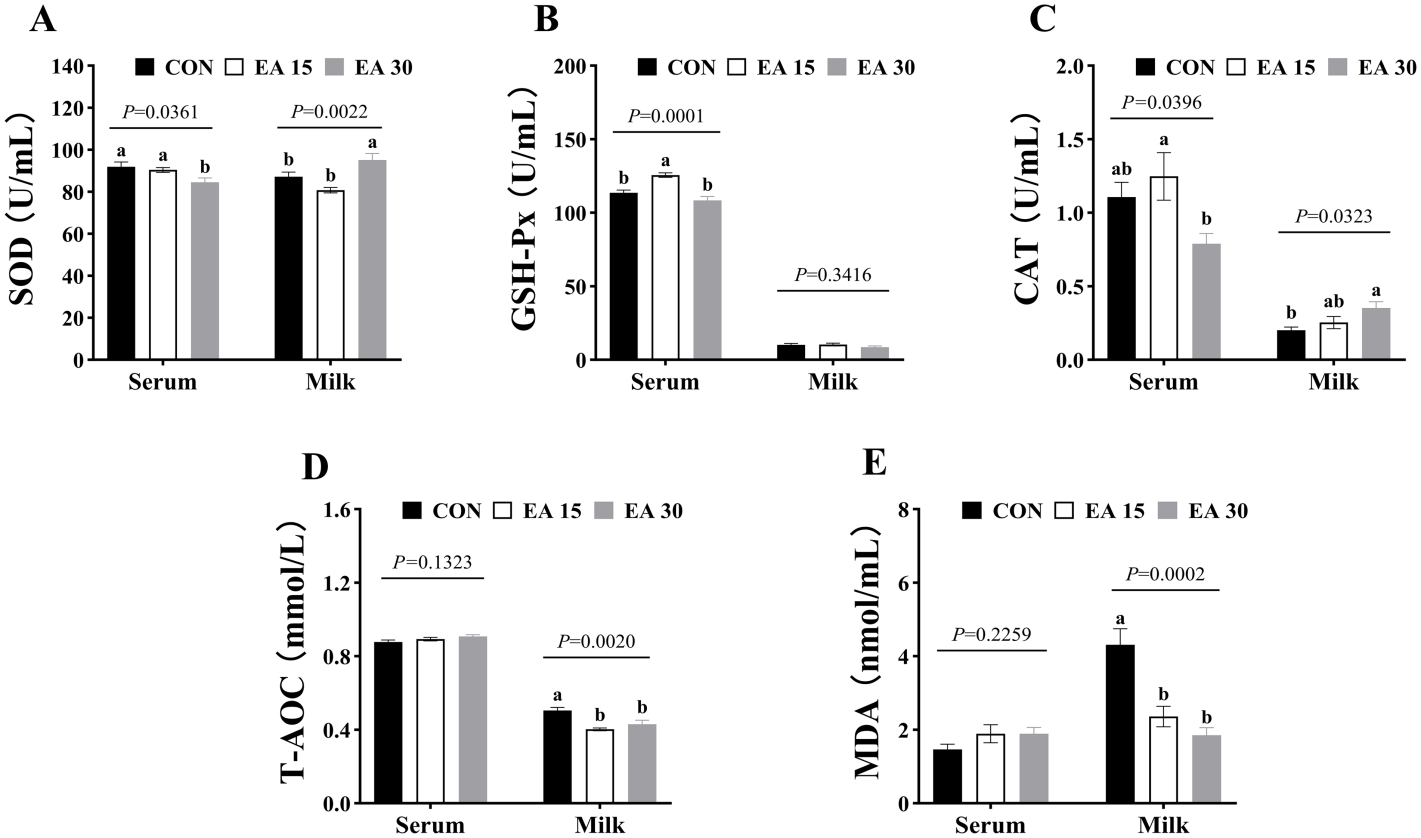
Effect of supplemental EA on antioxidant capacity in serum and milk of lactating mares on day 30 of the experiment. Effect of supplemental EA on serum and milk SOD activity in lactating mares **(A)**, serum and milk GSH-Px activity **(B)**, serum and milk CAT activity **(C)**, serum and milk T-AOC concentrations **(D)**, serum and milk MDA concentrations **(E)**. Values are means with their standard errors represented by vertical bars. Different letters in the same bar chart represent significant differences (^a,b^*p* < 0.05). SOD, superoxide dismutase; GSH-Px, glutathione peroxidase; CAT, catalase; T-AOC, total antioxidant capacity; MDA, malondialdehyde. CON: the control group, receiving no EA supplementation (*n* = 6); EA 15: the EA 15 group, each mare was supplemented with EA 15 mg/kg BW every day (*n* = 6); EA 30: the EA 30 group, each mare was supplemented with EA 30 mg/kg BW every day (*n* = 6).

Serum SOD activity was lower in the EA30 group mares than in the CON and EA15 groups mare (*p* = 0.0361); whereas milk SOD activity was higher in the EA30 group than in the other two groups (*p* = 0.0022; Figure 2A). Serum GSH-Px enzyme activity was higher in the EA15 group mares than in the CON and EA30 group mares (*p* = 0.0001; Figure 2B). Serum CAT activity was higher in the EA15 group mares than in the EA30 group mares (*p* = 0.0396), while milk CAT activity was higher in the EA30 group mares than in the CON group mares (*p* = 0.0323; Figure 2C). Milk T-AOC (*p* = 0.0020) and MDA (*p* = 0.0002) concentrations were lower in mares in both the EA15 and EA30 groups compared to mares in the CON group (Figures 2D,E).

### 3.4 Fecal pH and VFA in lactating mares

As shown in Table 4, EA supplementation had no significant effect on the mare’s fecal pH, acetate, propionate, isobutyrate, butyrate, isovalerate, valerate, and total volatile fatty acids (TVFA) content (*p* > 0.05). However, compared to the CON group, supplementation with 15 mg/BW/day EA showed numerical effects on the contents of acetate, propionate, butyrate, and TVFA in the feces of lactating mares.

**Table 4:** Effect of supplemental EA on fecal pH and VFA in lactating mares.

Items	Treatments			SEM	*p*-value
	CON	EA 15	EA 30		
pH	6.54	6.60	6.56	0.0760	0.8602
Acetate, mmol/L	22.20	29.95	24.94	2.4323	0.1068
Propionate, mmol/L	4.04	4.97	4.11	0.3878	0.2036
Isobutyrate, mmol/L	0.46	0.59	0.52	0.0463	0.1599
Butyrate, mmol/L	1.59	2.06	1.76	0.1947	0.2607
Isovalerate, mmol/L	0.82	1.11	0.99	0.1196	0.2482
Valerate, mmol/L	0.22	0.23	0.23	0.0112	0.5851
TVFA, mmol/L	29.33	38.92	32.54	3.1267	0.1216

CON: the control group, receiving no EA supplementation (*n* = 6); EA 15: the EA 15 group, each mare was supplemented with EA 15 mg/kg BW every day (*n* = 6); EA 30: the EA 30 group, each mare was supplemented with EA 30 mg/kg BW every day (*n* = 6). SEM, standard error of the mean.

### 3.5 OTU number and alpha diversity of fecal bacteria in lactating mares

For all mares, milk production peaked on day 30 of the experiment. Furthermore, compared with the mares in the CON group, the EA15 group had a more pronounced effect than the EA30 group on milk production, milk fat content, serum and milk hormone concentrations and antioxidant enzyme activities, and fecal TVFA content in lactating mares. Therefore, the study further investigated variations in fecal microbial diversity between mares in the EA15 and CON groups on day 30.

**Figure 3:**
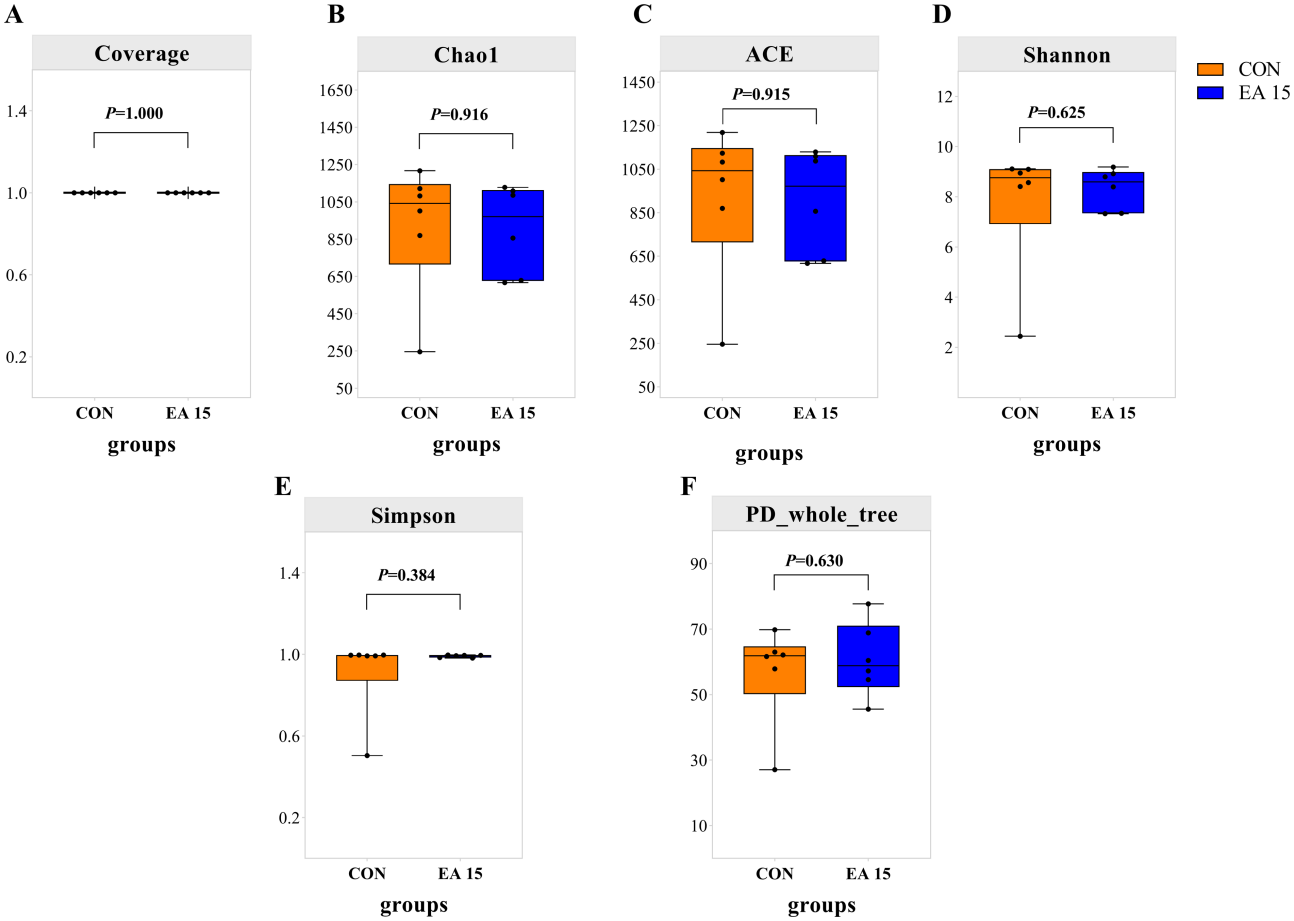
Effect of supplemental EA on the alpha diversity of fecal bacteria in lactating mares on day 30 of the experiment. **(A–F)** Alpha diversity metrics. **(A)** Coverage. **(B)** Chao1. **(C)** ACE. **(D)** Shannon. **(E)** Simpson. **(F)** PD_whole_tree indexes for fecal microbiota of lactating mares. Values are means with their standard errors represented by vertical bars. CON: the control group, receiving no EA supplementation (*n* = 6); EA 15: the EA 15 group, each mare was supplemented with EA 15 mg/kg BW every day (*n* = 6).

The number of OTUs shared by the CON group and the EA15 group was 1,074; the number of unique OTUs was 2,838 in the CON group, while the number of unique OTUs was 2,840 in the EA15 group. As shown in Figure 3, the coverage values for both groups were 0.99, indicating that the sequencing depth could accurately reflect the composition of the mare’s fecal bacteria (Figure 3A). EA15 supplementation had no statistically significant impact on the alpha diversity indices (Chao1, ACE, Shannon, Simpson, PD_whole_tree) of fecal bacteria compared to the mares in the CON group (*p* > 0.05), though numerical differences were observed (Figure 3). The Chao1 and ACE indices in the EA15 group (903.81; 905.02) were lower than in the CON group (922.64; 923.96) (Figures 3B,C), whereas the Shannon and Simpson indices in the EA15 group (8.33; 0.99) were higher than in the CON group (7.76; 0.91) (Figures 3D,E). The phylogenetic assessment based on PD_whole_tree (where higher values indicate greater community diversity) was higher in the EA15 group (60.71) than in the CON group (56.89) (Figure 3F).

### 3.6 Beta diversity of fecal bacteria in lactating mares

**Figure 4:**
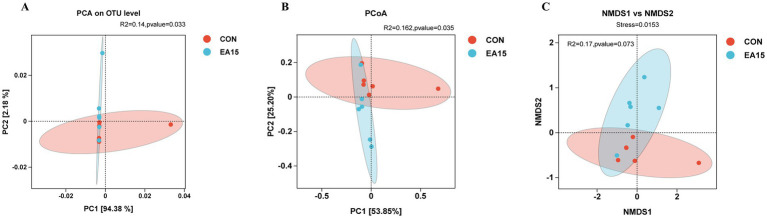
Effect of supplemental EA on the beta diversity of fecal bacteria in lactating mares. **(A–C)** Beta diversity metrics. **(A)** PCA based on the OTU level. Points of different colors or shapes represent different sample grouping situations, a shorter distance between sample points indicates greater similarity of bacteria (*n* = 6 for each group). **(B)** PCoA plot based on weighted UniFrac distances with PERMANOVA analysis. **(C)** NMDS plot based on weighted UniFrac distances. PCA, principal component analysis; PCoA, principal coordinate analysis; NMDS, nonmetric multidimensional scaling. CON: the control group, not supplemented with EA (*n* = 6); EA 15: the EA 15 group, each mare was supplemented with EA 15 mg/kg BW every day (*n* = 6).

As shown in Figure 4, PCA based on the OTU level of the EA15 and CON groups explained 94.38 and 2.18% in the PC1 and PC2 axes, respectively, which could adequately explain the variation between samples; the degree of difference in the composition of the bacterial flora between samples within the EA15 and CON groups was low (R^2^ = 0.14), whereas the difference in the diversity of the bacterial flora between the two groups was significant (PERMANOVA: *p* = 0.033) (Figure 4A). PCoA based on the weighted UniFrac distance algorithm explained 53.85 and 25.20% of the variance along the PC1 and PC2 axes, respectively, demonstrating distinct clustering within the EA15 and CON groups. Small differences in the composition of the bacterial communities between samples within the groups (*R*^2^ = 0.162), and significant differences in the diversity of the bacterial communities between the two groups (PERMANOVA: *p* = 0.035) (Figure 4B). The results of the NMDS based on the Weighted Unifrac distance algorithm showed that Stress = 0.0153 between the EA15 group and the CON group could accurately reflect the degree of difference between the samples, but there was no significant difference in the composition of the bacterial flora between the two groups (PERMANOVA: *p* = 0.073) (Figure 4C).

### 3.7 Relative abundance of fecal bacteria at different taxonomic levels in lactating mares

**Figure 5:**
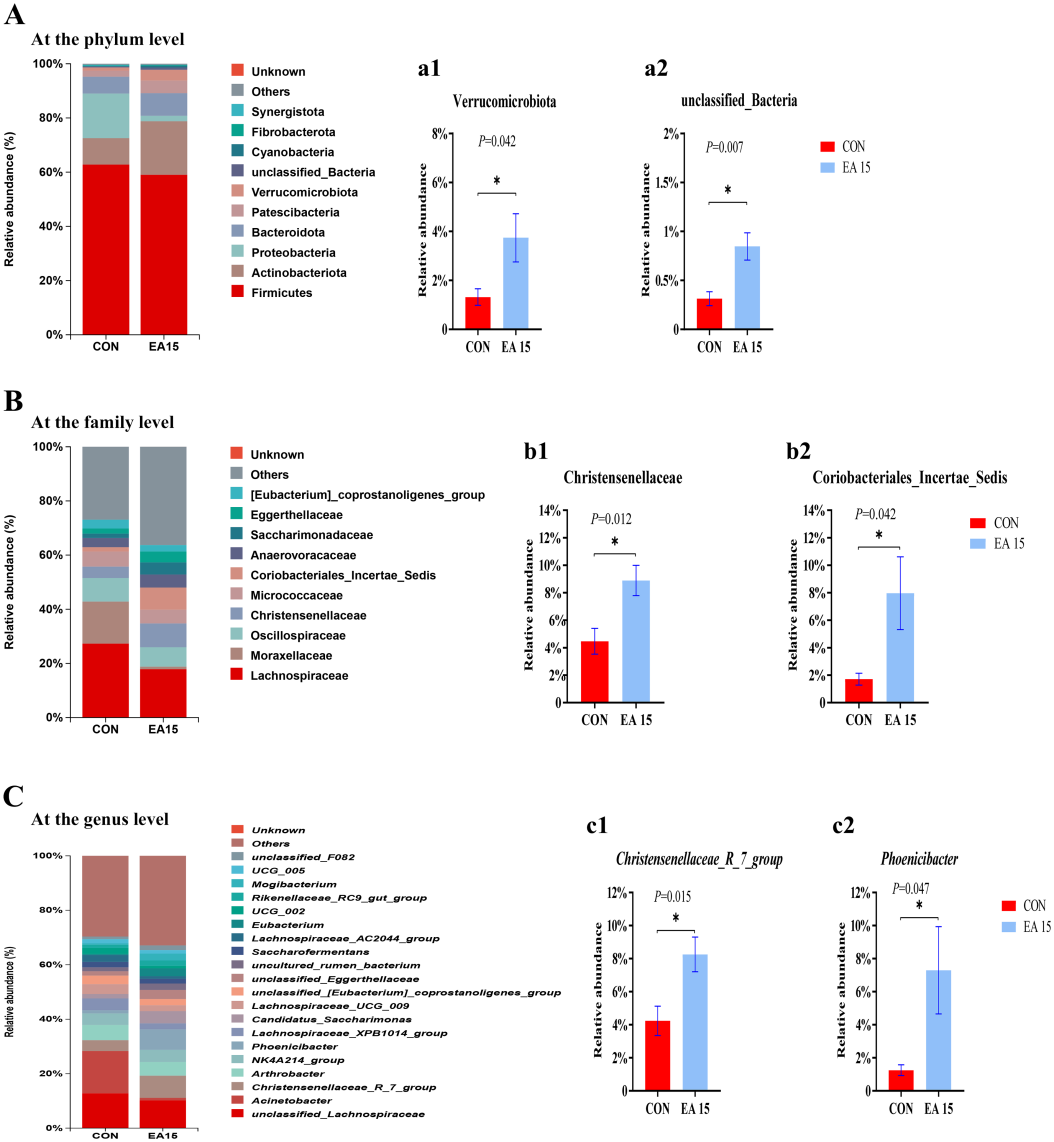
Effect of supplemental EA on the relative abundance of fecal bacteria at different taxonomic levels in lactating mares on day 30 of the experiment. **(A)** Microbiota composition at the phylum level. **(a1,a2)** Column plots indicate that the relative abundance of bacteria at the phylum level was significantly different between the CON and EA15 groups. **(B)** Microbiota composition at the family level. **(b1,b2)** Column plots indicate that the relative abundance of bacteria at the family level was significantly different between the CON and EA15 groups. **(C)** Microbiota composition at the genus level. **(c1,c2)** Column plots indicate that the relative abundance of bacteria at the genus level was significantly different between the CON and EA15 groups. For **a1–c2**, statistical significance was determined using *t*-test, and values are means with their standard errors represented by vertical bars. ^*^p < 0.05. CON: the control group, receiving no EA supplementation (*n* = 6); EA 15: the EA 15 group, each mare was supplemented with EA 15 mg/kg BW every day (*n* = 6).

The relative abundances of the top 10 bacterial phyla are presented in Figure 5A. Firmicutes, Actinobacteriota, and Proteobacteria predominated in the CON group, whereas Firmicutes, Actinobacteriota, and Bacteroidota were the dominant phyla in the EA15 group, collectively accounting for over 80% of the total bacterial relative abundance. The relative abundance of the Actinobacteriota was higher in the EA15 group (19.74%) than in the CON group (9.75%) (*p* > 0.05). The relative abundance of the Proteobacteria was lower in the EA15 group (2.02%) than in the CON group (16.46%) (*p* > 0.05). The relative abundance of Verrucomicrobiota and unclassified_Bacteria was significantly higher in the EA15 group than in the CON group (*p* = 0.042, *p* = 0.007) (Figures 5a1,a2).

The relative abundances of the top 10 species at the family level are shown in Figure 5B. The dominant bacteria in the CON group were Lachnospiraceae, Moraxellaceae, and Oscillospiraceae. The dominant bacteria in the EA15 group were Lachnospiraceae, Oscillospiracea, Christensenellaceae, and Coriobacteriales_Incertae_Sedis. The relative abundance of Moraxellaceae was lower in the EA15 group (0.97%) than in the CON group (15.53%) (*p* > 0.05). The relative abundance of Christensenellaceae and Coriobacteriales_Incertae_Sedis was significantly higher in the EA15 group than in the CON group (*p* = 0.012, *p* = 0.042) (Figures 5b1,b2).

The relative abundances of the top 10 species at the genus level are shown in Figure 5C. The dominant bacteria in the CON group were *unclassified_Lachnospiraceae*, *Acinetobacter*, and *Arthrobacter*. The dominant bacteria in the EA15 group were *unclassified_Lachnospiraceae*, *Christensenellaceae_R_7_group*, and *Phoenicibacter*. The relative abundance of *Acinetobacter* was lower in the EA15 group (0.92%) than in the CON group (15.53%) (*p* > 0.05). The relative abundance of *Christensenellaceae_R_7_group* and *Phoenicibacter* was significantly higher in the EA15 group than in the CON group (*p* = 0.015, *p* = 0.047) (Figures 5c1,c2).

### 3.8 Linear discriminant analysis coupled with effect size analysis

**Figure 6:**
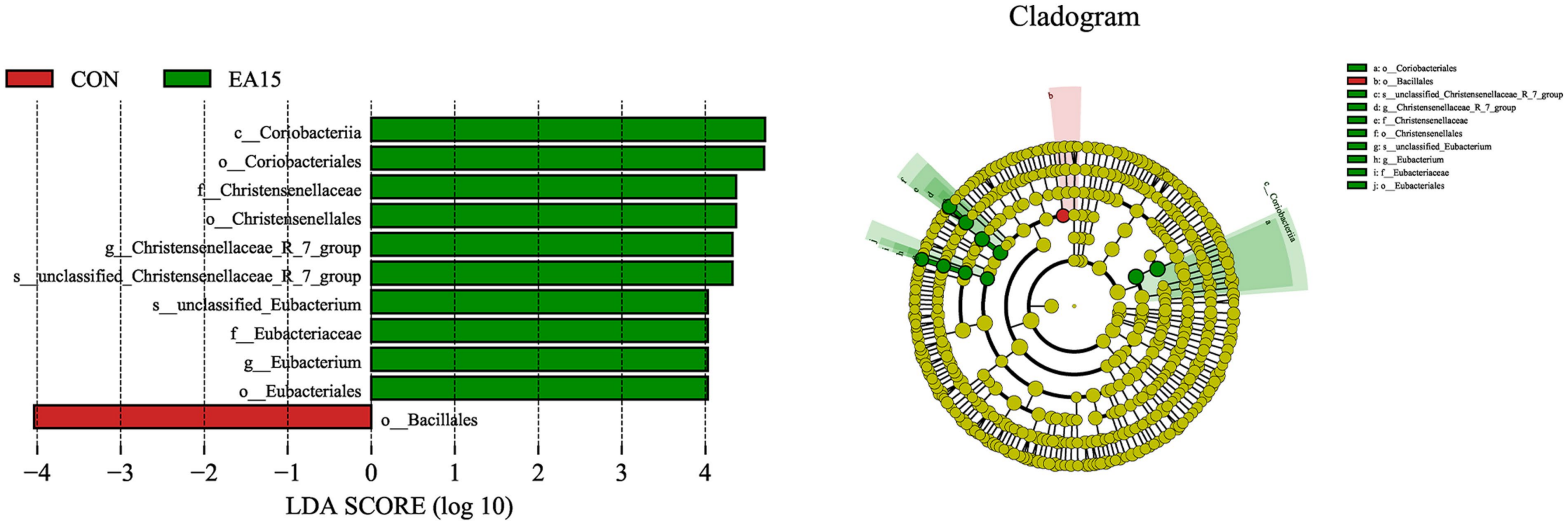
Effect of supplemental EA on the LEfSe of fecal bacteria in lactating mares on day 30 of the experiment. Linear discriminant analysis (LDA) distribution, and the score = 4 means significant. Cladogram of LEfSe shows taxonomic profiling from the phylum to species level, the yellow node represents no difference, but other color nodes represent significant difference. CON: the control group, receiving no EA supplementation (*n* = 6); EA 15: the EA 15 group, each mare was supplemented with EA 15 mg/kg BW every day (*n* = 6).

The LEfSe analysis was used to find species that were statistically different between groups at different taxonomic levels. As shown in Figure 6, 11 species were significantly different between the CON and EA15 groups at different taxonomic levels. The only species with significant differences in the CON group were Bacillales. There were 10 species with significant differences in the EA15 group, namely Coriobacteriia, Coriobacteriales, Christensenellaceae, Christensenellales, *Christensenellaceae_R_7_group*, *unclassified_Christensenellaceae_R_7_group*, *unclassified_Eubacterium*, Eubacteriaceae, Eubacterium, and Eubacteriales.

### 3.9 Fecal bacterial function prediction in lactating mares

**Figure 7:**
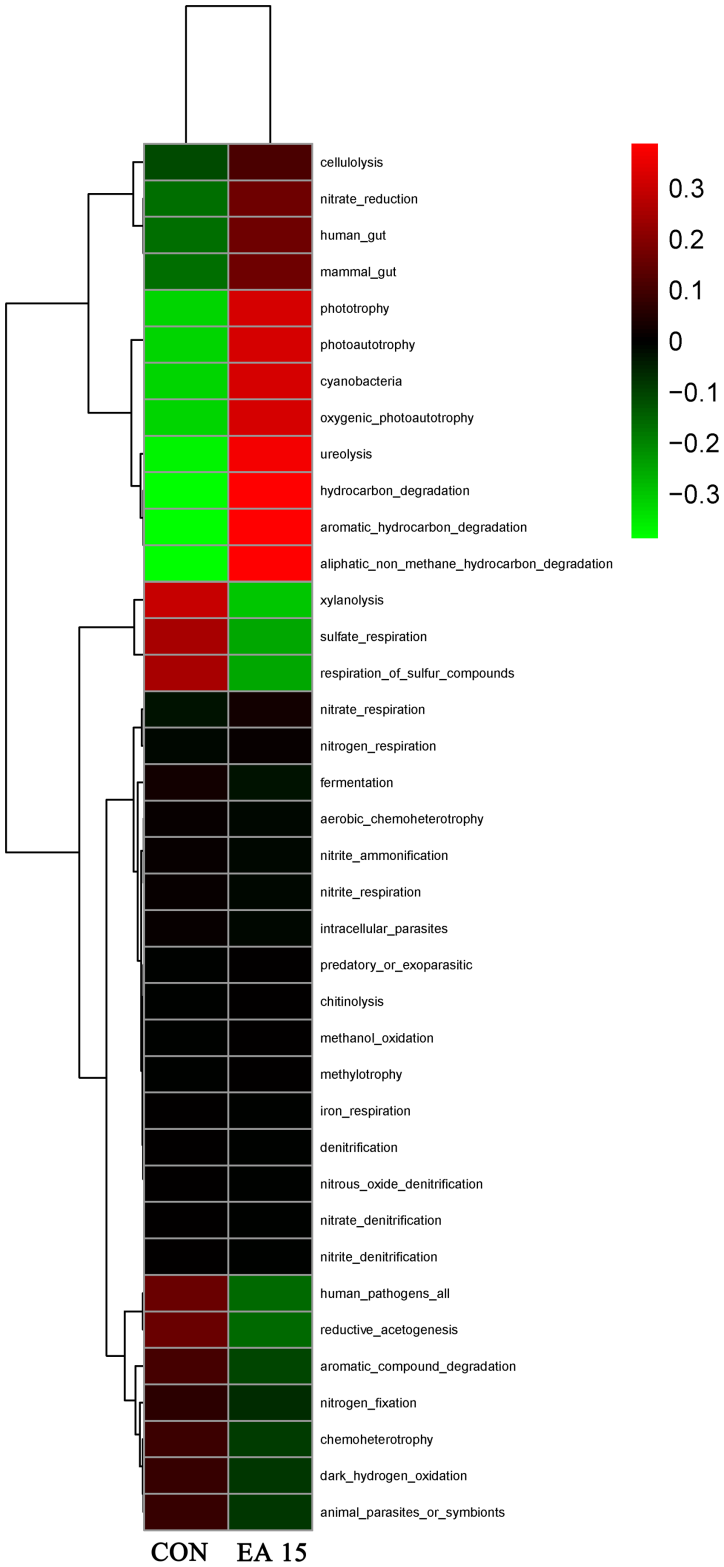
Effect of supplemental EA on fecal bacterial function prediction in lactating mares on day 30 of the experiment. Tax4Fun functional clustering heatmap prediction of fecal microbiota. Red denotes higher enrichment while green denotes lower enrichment. CON: the control group, receiving no EA supplementation (*n* = 6); EA 15: the EA 15 group, each mare was supplemented with EA 15 mg/kg BW every day (*n* = 6).

Prediction of potential functions of the fecal microbial community based on Tax4Fun predicted a total of 38 functions. As shown in Figure 7, The EA15 group exhibited positive correlations with 12 functions, including cellulolysis, nitrate_reduction, human_gut, mammal_gut, phototrophy, photoautotrophy, cyanobacteria, oxygenic_photoautotrophy, ureolysis, hydrocarbon_degradation, aromatic_hydrocarbon_degradation, and aliphatic_non_methane_hydrocarbon_degradation, while showing negative correlations with sulfate_respiration, respiration_of_sulfur_compounds, human_pathogens_all, and reductive_acetogenesis. In contrast, the CON group displayed an inverse pattern, positively correlating with the latter five functions while negatively associating with the 12 functions enriched in the EA15 group.

### 3.10 Spearman’s correlation analysis between indicators

**Figure 8:**
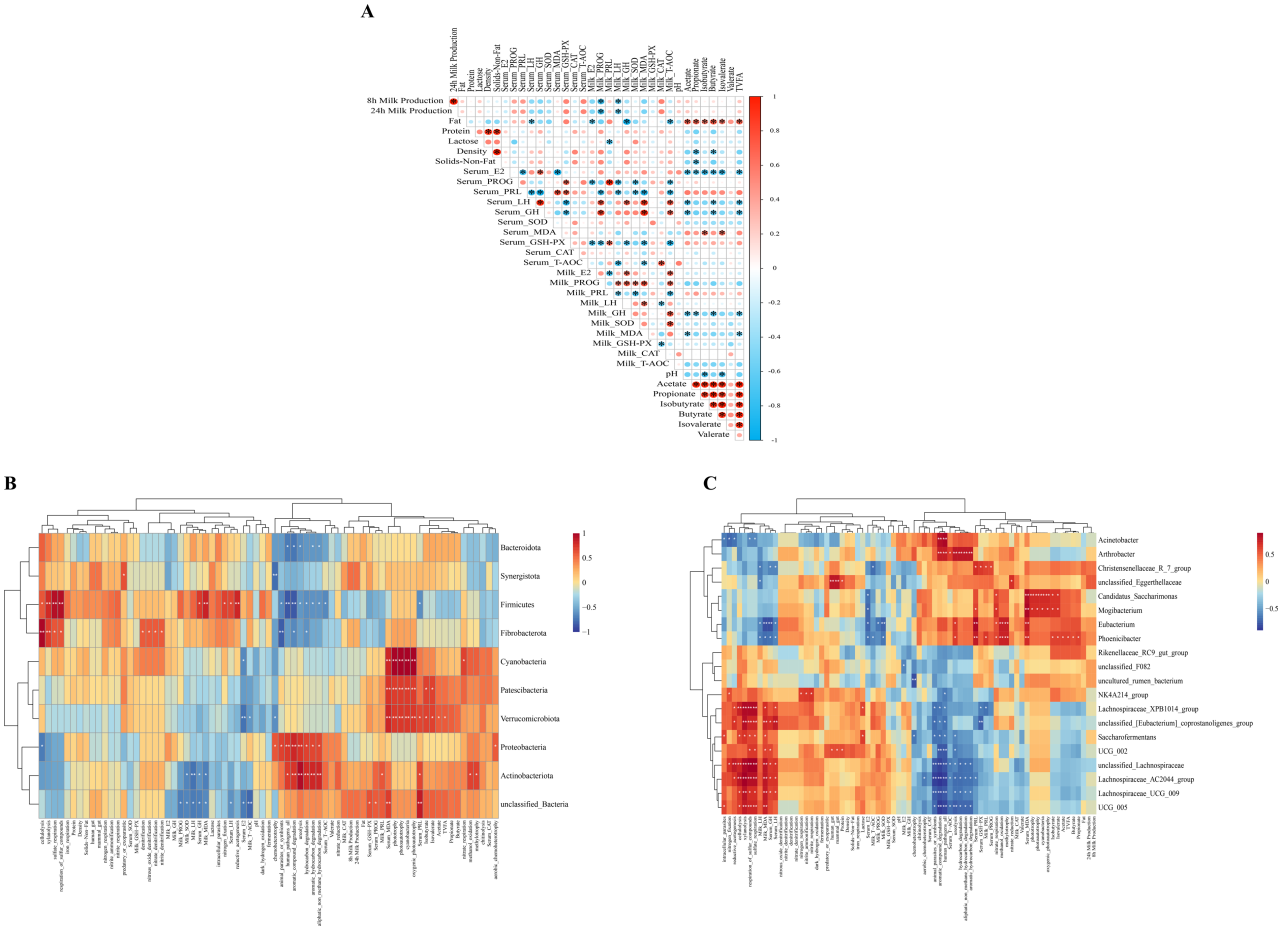
Spearman’s correlation analysis between indicators. **(A)** Spearman’s correlation analysis mare milk production, milk composition, serum hormones and antioxidant capacity, milk hormones and antioxidant capacity and fecal fermentation parameters. Heatmap of Spearman’s correlations among mare milk production, milk composition, serum hormones and antioxidant capacity, milk hormones and antioxidant capacity, fecal fermentation parameters and fecal bacterial prediction function, and differential fecal microbiota at the phylum **(B)**, and genus **(C)** levels. The red and blue panes represent positive and negative correlations, respectively. Color intensity indicates the Spearman’s *r*-value of correlations in each panel. The asterisks indicate significant correlations (^*^p < 0.05 and ^**^p < 0.01).

The results of Spearman’s correlation analysis between key indicators are shown in Figure 8. Mare milk production at 8 h and 24 h was negatively correlated with PROG and LH hormones in milk (*p* < 0.05). Fat in mare’s milk was negatively correlated with serum LH, milk E2, GH concentrations, and milk T-AOC levels (*p* < 0.05), whereas it was positively correlated with fecal contents of acetate, propionate, isobutyrate, butyrate, isovalerate and TVFA (*p* < 0.05). Serum MDA levels correlated positively with fecal isobutyrate and isovalerate contents (*p* < 0.05). Milk MDA levels were negatively correlated with fecal acetate and TVFA contents (*p* < 0.05). Fecal pH was negatively correlated with fecal isobutyrate and isovalerate (*p* < 0.05). Different types of short-chain fatty acids in feces were positively correlated as well as TVFA content (*p* < 0.05), with the exception of valerate (Figure 8A).

At the phylum level, Bacteroidota was negatively correlated with the aromatic_compound_degradation, human_pathogens_all, ureolysis, aliphatic_non_methane_hydrocarbon_degradation, and aromatic_hydrocarbon_degradation functions (*p* < 0.05). Verrucomicrobiota were positively correlated with serum PRL concentrations, fecal contents of acetate, isobutyrate, isovalerate and TVFA, oxygenic_photoautotrophy, cyanobacteria, photoautotrophy, phototrophy functions (*p* < 0.05 or *p* < 0.01), and negatively correlated with serum E2 levels, milk T-AOC activity, chemoheterotrophy functions (*p* < 0.05 or *p* < 0.01). Proteobacteria were positively correlated with the aerobic_chemoheterotrophy, aliphatic_non_methane_hydrocarbon_degradation, aromatic_hydrocarbon_degradation, hydrocarbon_degradation, ureolysis, aromatic_compound_degradation, human_pathogens_all, animal_parasites_or_symbionts, and chemoheterotrophy functions (*p* < 0.05 or *p* < 0.01). Actinobacteriota were positively correlated with serum PRL, milk PRL concentrations, methylotrophy, methanol_oxidation, aliphatic_non_methane_hydrocarbon_degradation, aromatic_hydrocarbon_degradation, hydrocarbon_degradation, ureolysis, aromatic_compound_degradation, human_pathogens_all functions (*p* < 0.05 or *p* < 0.01), and negatively correlated with milk LH and MDA levels (*p* < 0.05 or *p* < 0.01) (Figure 8B).

At the genus level, *Acinetobacter* positively correlated with the human_pathogens_all, aromatic_compound_degradation functions (*p* < 0.01), while showing negatively correlated with intracellular_parasites, nitrogen_fixation, reductive_acetogenesis, respiration_of_sulfur_compounds, sulfate_respiration functions (*p* < 0.05). *Christensenellaceae_R_7_group* was positively correlated with serum PRL concentrations, GSH-Px activity and milk PRL concentrations (*p* < 0.05), while negatively correlating with serum LH, GH, and milk LH concentrations (*p* < 0.05). *Phoenicibacter* was positively correlated with fecal acetate, propionate, isobutyrate, butyrate, isovalerate and TVFA contents, as well as with serum PRL, milk PRL concentrations, methylotrophy, methanol_oxidation, nitrate_respiration, ureolysis functions (*p* < 0.05 or *p* < 0.01). Conversely, *Phoenicibacter* negatively correlated with milk LH and MDA levels, as well as serum E2, LH, and GH concentrations (*p* < 0.05) (Figure 8C).

## 4 Discussion

Milk production and nutrient composition in mares are influenced by various factors, including breed, parity, lactation duration, nutritional intake, and udder health (Miraglia et al., 2020; Auclair-Ronzaud et al., 2022). Among these, dietary nutrition and lactation stage are the main factors influencing milk production and composition in mares of the same breed and similar parity (Centoducati et al., 2012; Markiewicz-Kęszycka et al., 2015; Barłowska et al., 2023). Pyles et al. (2020) reported that mares fed a high nonstructural carbohydrate (NSC) concentrate enhanced milk carbohydrate synthesis and daily milk production compared to mares fed a low NSC concentrate. In this study, EA supplementation increased the milk production of mares at 60 and 90 days of the experimental period. However, as a natural plant polyphenol compound, EA does not have the function of directly provide energy-based nutrients to enhance production performance. Nevertheless, EA can indirectly enhance performance through multiple synergistic pathways, such as improving the body’s oxidative state, regulating hormonal endocrine, and modulating the gut microbiota (Baradaran Rahimi et al., 2020; Qin et al., 2022). In this study, EA supplementation increased serum and milk PRL concentrations in lactating mares and reduced milk MDA concentration on the 30th day of the experiment. Therefore, EA may indirectly increase the milk yield of lactating mares by improving the oxidative state of mares and regulating hormones. On day 30 of this study, milk production of mares in all experimental groups reached peak levels, consistent with the physiological pattern of peak lactation in mares (typically occurring 2–3 months post-foaling) (Włodarczyk-Szydłowska et al., 2005; Pieszka et al., 2016). This may explain why EA supplementation at this stage did not exert significant effects; thus, there was no statistically significant difference in milk production between the CON group mares and the EA supplemented mares at this time point. However, long-term EA supplementation may produce a cumulative effect over time in the body. Based on this, it is reasonably deduced that EA supplementation to mares on days 60 and 90 of the experimental period may also enhance milk production by mitigating the body’s oxidative state and increasing the PRL concentration. Over the entire experimental period, EA supplementation appeared to help extend the duration of high milk production in lactating mares; compared with the CON group, mares in the EA supplemented group maintained relatively high milk production beyond peak lactation (on days 60 and 90 of the experiment).

In this study, milk production and composition, including fat, protein, lactose, density, and solids-non-fat of lactating mares exhibited a declining trend as lactation progressed. On day 30 of the experiment, milk production of mares across all groups was higher than on days 0, 60, and 90 of the experiment. Previous studies have shown that peak lactation occurs within 2–3 months postpartum, followed by a gradual decline, a pattern consistent with the findings of this study, as day 30 of the experiment coincided with the second month of lactation in the mares (Włodarczyk-Szydłowska et al., 2005; Pieszka et al., 2016). Comparative analyses with other breeds further contextualize these results. Murgese breed (light horses) produced 14 kg of milk per day, Tiro Pesante Rapido (heavy horses) 22 kg per day (Salimei and Fantuz, 2012), Lipizzan mares (warm-blooded horses) 8.24 kg per day on average (Kaić et al., 2019), and Lusitano horses 12.4 kg per day on average (Santos and Silvestre, 2008). In this study, the estimated daily milk yield of Yili mares on day 30 of the experiment ranged from 9.47 to 10.67 kg, aligning closely with previously reported values. Milk composition undergoes dynamic changes throughout lactation (Barreto et al., 2019). Czyżak-Runowska et al. (2021) observed significant declines in lactose (6.33 vs. 6.19), protein (2.25 vs. 1.84), and dry matter (9.65 vs. 9.28) in Polish Coldblood mares between weeks 10 and 25 of lactation. Similarly, Markiewicz-Kęszycka et al. (2015) reported reductions in fat (15.9 vs. 4.3), protein (22.5 vs. 16.7), and energy content (2.16 vs. 1.64) in Thoroughbred and Polish Half-Bred mares between days 28 and 180 of lactation. Pietrzak-Fiećko et al. (2013) noted that the proportion of saturated fatty acids in Polish Coldblood mares increased in late lactation (4–6 months) compared to mid (2–4 months) and early lactation (0–2 months), whereas unsaturated fatty acid content followed an inverse pattern. Consistent with these findings, in this study, the protein, lactose, density, and solids-non-fat contents of the mares milk on days 0 and 30 of the experiment were all higher than those on days 60 and 90. However, milk fat content peaked on day 60, surpassing levels recorded on days 0, 30, and 90 of the experiment. These results are similar to trends observed in previous studies.

Hormones play an important role in the lactation process, and the occurrence and maintenance of lactation depend mainly on regulating the nervous and endocrine systems (Akers, 2006, 2017). The lactation process is orchestrated by a complex network of hormonal interactions, including estrogen, PROG, LH, GH, cortisol, PRL, insulin and others (Pal et al., 2019). These hormones can be broadly categorized into 2 groups: lactation-promoting hormones, such as PRL, E2, and GH, and lactation-inhibiting hormones, such as PROG (Kobayashi et al., 2016; Gavelis et al., 2018). Variations in endocrine hormone levels influence lactation to differing extents (Macrina et al., 2011). PRL, GH, and E2 play a crucial role in the synthesis of main milk components, the proliferation and differentiation of mammary epithelial cells, and the maintenance of lactation (Ni et al., 2021). Research has shown that factors such as physiological state, diet, environment (humidity, temperature, light, season), and stress all affect the nervous and endocrine systems, which in turn affect hormone production (Digiovanni et al., 2015; Li et al., 2019; Li et al., 2021). Integrating the above results of milk production and composition, milk production and the concentrations of most milk components (excluding fat) peaked on day 30 of the trial. Consequently, this study focused on evaluating the effects of EA supplementation on serum and milk hormone concentrations in mares at this time point. Specifically, EA supplementation increased serum and milk PRL concentrations while reducing serum LH and milk GH concentrations in lactating mares on day 30 of the study. EA possesses structural characteristics that enable the endocrine system to interfere, exhibiting androgen agonist, anti-thyroid hormone, and estrogenic activities. Its metabolites, urolithins (Uro-A and Uro-B), share structural similarities with estradiol and are considered potential phytoestrogens (Zhang et al., 2023; Skledar et al., 2019). Tong et al. (2018) demonstrated that supplementation with exogenous estrogen (17β-estradiol) can increase PRL concentrations in the serum and milk of lactating Holstein cows, findings similar to those of the present study. Given the estrogenic activity of EA and its metabolite urolithin possess, they may increase PRL concentrations in the serum and milk of lactating mares. During pregnancy and lactation, elevated PRL levels suppress gonadotropin-releasing hormone secretion in the hypothalamus, thereby inhibiting LH and follicle-stimulating hormone production (Donato and Frazão, 2016; Cocks Eschler et al., 2018). This may be the potential reason for the regulation of changes in hormone concentrations in serum and milk of lactating mares following EA supplementation in this study. Jing et al. (2024) reported that LH concentrations in the serum and milk of lactating Yili mares grazing on grasslands ranged from 1 to 3 mIU/mL. Although EA supplementation in this study reduced LH concentrations in lactating mares, LH concentrations measured in this study for all groups remained in the ranges of previous observations. Beyond its role in mammary gland development and lactation, GH is a key regulator of growth and development (Steyn, 2015). Despite the reduction in milk GH concentrations observed in this study, however, a previous study by He et al. (2024) from our team indicated that, compared to the control group, supplementing lactating mares with EA did not affect the average daily weight gain of foals (CON: 0.41 kg/d; EA15: 0.47 kg/d; EA30: 0.45 kg/d) or total height growth (CON: 18.67 cm; EA15: 23.28 cm; EA30: 22.50 cm).

During peak lactation, the rapid development of the mammary glands and the synthesis of large quantities of milk increases the oxygen-demanding metabolic activity of the mammary epithelial cells, which generates large amounts of reactive oxygen radicals and induces oxidative stress. This oxidative stress not only compromises milk production and quality but also negatively impacts the health of the dam (Berchieri-Ronchi et al., 2011; Sordillo, 2013). Enhancing antioxidant capacity during this critical period is essential for optimizing lactation performance. As a natural polyphenolic antioxidant, EA effectively scavenges free radicals, inhibits lipid peroxidation, enhances antioxidant enzyme activity, and reduces the accumulation of peroxides, thereby strengthening systemic antioxidant defenses. Its metabolite, urolithin, further contributes to sustained oxidative stress mitigation through free radical scavenging cascade reactions (Galano et al., 2014; Kilic et al., 2014). Gul et al. (2022) demonstrated that supplemental EA was able to increase serum and liver SOD and GSH-Px activities while reducing MDA levels in yellow-brown laying hens compared to the control group. Similarly, Xiao et al. (2022) reported that EA supplementation in piglets with paraquat-induced intestinal injury enhanced serum SOD activity, lowered MDA levels, alleviated oxidative stress, restored intestinal morphology, and reduced intestinal barrier permeability. In this study, EA supplementation enhanced GSH-Px and CAT activity in serum (EA 15 group) and SOD and CAT enzyme activity in the milk (EA30 group) of lactating mares. Additionally, it reduced T-AOC and MDA levels in milk. While the simultaneous decline in milk T-AOC and MDA levels appears contradictory, this discrepancy may be attributed to the limited sample size or inter-individual variability among mares in this study. Nonetheless, the observed increases in milk SOD and CAT activities, along with the reduction in MDA levels, suggest that EA supplementation may alleviate or prevent oxidative stress in the mammary glands of lactating mares. The antioxidant enzyme activity observed in the serum of lactating mares in this study were similar to findings from previous research. This effect may be attributed to both the intrinsic molecular structure of EA and its regulatory influence on key signaling pathways. Structurally, EA contains 2 lactone and 4 hydroxyl groups, with the 4 phenolic hydroxyls serving as the primary sites for interactions with reactive oxygen and nitrogen species. These hydroxyl groups donate hydrogen atoms to neutralize oxygen free radicals, thereby reducing oxidative stress and enhancing systemic antioxidant capacity (Tiwari and Mishra, 2013; Mişe Yonar et al., 2014; Nugroho et al., 2014). At the mechanistic level, EA has been shown to mitigate reactive oxygen species production and upregulate SOD activity by inhibiting the phosphatidylinositol 3-kinase /protein kinase B signaling pathway. Additionally, it enhances the expression of glutathione peroxidase 1 via activation of the nuclear factor erythroid 2-related factor 2-mediated pathway, further strengthening antioxidant defense mechanisms (Lin et al., 2021; Zhang et al., 2022). Whether the antioxidant effects of EA in lactating mares observed in this study are mediated through phosphatidylinositol 3-kinase/protein kinase B and nuclear factor erythroid 2-related factor 2 pathways warrants further investigation. Furthermore, studies have shown that short-chain fatty acids (SCFAs) can regulate the expression of antioxidant enzymes. Huang et al. (2017) reported that the addition of acetate and butyrate inhibited oxidative stress in mouse glomerular mesangialn cells induced by high glucose and lipopolysaccharide and increased the activity of antioxidant enzymes in the cells. In this study, EA supplementation elevated fecal acetate, butyrate, and TVFA contents in lactating mares. Moreover, correlation analysis revealed a significant negative association between milk MDA levels and fecal acetate and TVFA contents, consistent with the findings of Huang et al. (2017). Based on these results, it is reasonable to infer that EA may enhance antioxidant enzyme activity by modulating SCFA production in the gut of lactating mares.

SCFAs are one of the important metabolites of intestinal microorganisms, which are closely related to the energy metabolism of animals and can promote the development of the animal’s intestinal tract, improve immune function, and protect animal health by regulating the animal’s intestinal energy, intestinal barrier and body metabolism (Sadler et al., 2020; van der Hee and Wells, 2021). In ruminants, SCFAs, as the main product of rumen fermentation, provide approximately 75% of the body’s energy (Shen et al., 2016). In non-ruminant equids, SCFAs produced in the hindgut (cecum and colon) account for approximately 30% of the energy requirements under maintenance conditions (National Research Council, 2007). Previous studies have demonstrated that EA supplementation can increase the content of SCFAs in the cecal contents of 3-week-old young mice and yellow-feathered broilers (Duan et al., 2022; Wang F. et al., 2022). In this study, while EA supplementation did not significantly alter fecal levels of acetate, propionate, isobutyrate, butyrate, isovalerate, or TVFA in lactating mares, all of these were elevated compared to the control group of mares. This result may be attributed to the limited sample size (*n* = 6/group) or individual variability among mares in this study. Our *post hoc* power analysis using G*Power software (version 3.1.9.7; https://www.psychologie.hhu.de/arbeitsgruppen/allgemeine-psychologie-und-arbeitspsychologie/gpower) for the Omnibus one-way ANOVA among the three groups revealed a low statistical power of 26% for detecting a medium effect size (*f* = 0.4). For the primary comparison (CON vs. EA15 groups), an independent t-test with the observed effect size (effect size *d* = 0.8) yielded only 24% power at *α* = 0.05 (two-tailed), which is below the recommended 80% threshold, thereby increasing the risk of a type II error. These results indicate that although EA supplementation may exhibit a biological trend toward increased SCFA production, further studies with larger sample sizes and longer durations are needed to confirm these effects. However, in practical horse farming, recruiting large numbers of animals while meeting the necessary experimental conditions is often challenging. SCFAs can be absorbed by intestinal epithelial cells and undergo β-oxidation through the hydroxymethylglutaryl-coenzyme A cycle, producing glucose that provides energy for the body (Byrne et al., 2015). Therefore, this may also be one of the reasons why the mares in the EA supplementation group produced more milk than the control group throughout the experimental period in this study. SCFAs are not only directly involved in the ab initio synthesis of fatty acids in the mammary gland as precursors to milk fat synthesis in ruminants, but also act as signaling molecules that regulate mammary fatty acid metabolism and milk fat synthesis (He et al., 2020). In this study, a significant positive correlation was found between milk fat content and fecal levels of acetate, propionate, butyrate, and TVFA. However, the mechanisms and pathways involved in milk fat synthesis in mares remain unclear. Therefore, further investigation is needed to determine whether SCFAs serve as precursors for milk fat synthesis and how they might act as signaling molecules to regulate this process in lactating mares.

Gut microorganisms play a pivotal role in digestion, nutrient absorption, and the regulation of host metabolism and health (Ringseis et al., 2020). Similarly, fecal microorganisms share similarities in composition, relative abundance, and function with gut microbes, contributing significantly to gut structure and nutrient processing (Park et al., 2014). Studies have shown that gut microbiota is a key factor influencing the bioactivity of polyphenols, with a bidirectional interaction between polyphenols and gut flora. Polyphenols can modulate the composition and function of gut microorganisms (a “probiotic effect”), while gut microbes metabolize polyphenols into biologically active, low molecular weight derived metabolites (Liu et al., 2020; Wang X. et al., 2022). EA as a polyphenols, gut microbes may be a target for the action of EA (Guan et al., 2017). Since the beneficial effects of EA supplementation were manifested differently in milk production, milk fat content, serum and milk hormone concentrations and antioxidant enzyme activities, and fecal TVFA content of lactating mares, and were mainly observed in the EA 15 group, we assessed the effect of EA on fecal microbial composition in the CON and EA 15 groups of mares. In this study, although there was no significant difference in microbial α diversity between the 2 groups, the diversity indices of fecal bacteria in mares of the EA15 group were higher than those in the CON group mares. This indicates that EA may enhance fecal microbial diversity and richness in lactating mares. Beta diversity analysis showed a significant clustering of the fecal microbial community structure between the EA 15 group and CON group, indicating that EA supplementation altered the gut microbiota composition. Further analysis at the phylum level showed that EA supplementation increased the relative abundance of Actinobacteriota and Bacteroidota, significantly elevated the relative abundance of Verrucomicrobiota and unclassified bacteria, and reduced the abundance of Proteobacteria. Previous studies have indicated that Actinobacteriota plays a role in maintaining gut homeostasis and supporting the immune system (Binda et al., 2018). In addition, Actinobacteria improve the digestion and absorption of nutrients in the host by secreting endogenous enzymes such as cellulase, chitinase, xylanase, and pectinase, as well as producing extracellular enzymes like amylase and protease (Hashemipour et al., 2016; Ma et al., 2018). By colonizing on the surface of the intestinal mucosa, Bacteroides can promote the production of nutrients and energy required by the host body, and participate in many important metabolic activities, including carbohydrates fermentation, utilization of nitrogenous substances, and the biotransformation of bile acids and other steroids. It also plays a protective role by resisting the attachment of invasive intestinal pathogens (Marcobal et al., 2011; Tamana et al., 2021). Verrucomicrobia can participate in host organism immune regulation and enhance glucose metabolism, and is considered a potential biomarkers of gut health (Anderson et al., 2017; Fujisaka et al., 2020). Studies have shown that Verrucomicrobia may help maintain immune homeostasis by upregulating the transcription factor foxp3 in regulatory T cells (Lindenberg et al., 2019). Conversely, the abundance of Proteobacteria is positively correlated with intestinal diseases and is the iconic flora of intestinal flora imbalance (Zhu et al., 2019). Correlation analysis in this study revealed that Actinobacteria was positively correlated with mare serum and milk PRL levels, and negatively correlated with milk MDA levels. Verrucomicrobia was positively correlated with mare serum PRL levels and fecal acetate and TVFA contents. Proteobacteria were positively correlated with human_pathogens_all, animal_parasites_or_symbionts functions, and negatively correlated with cellulolysis function. In summary, EA supplementation may improve the gut microbiota of lactating mares by promoting the abundance of beneficial bacteria and inhibiting the proliferation of harmful microorganisms, thereby enhancing gut health, nutrient absorption, and anti-inflammatory capacity.

In this study, EA supplementation in lactating mares decreased the relative abundance of Moraxellaceae, and significantly increased the relative abundance of Christensenellaceae and Coriobacteriales_Incertae_Sedis at the family level. At the genus level, the relative abundance of *Acinetobacter* decreased, while the relative abundance of *Christensenellaceae_R_7_group* and *Phoenicibacter* significantly increased. Moraxellaceae includes genera such as *Acinetobacter*, *Moraxella* (the type genus), *Psychrobacter*, and the more recently proposed *Alkanindiges*, *Paraperlucidibaca*, and *Perlucidibaca*. The family includes species that colonize mucosal membranes or skin and can sometimes cause a variety of infections (Teixeira and Merquior, 2014). Among these, *Acinetobacter* can lead to systemic inflammation by inducing high levels of interleukin-6, interferon-γ, and other expressions (Chen et al., 2020). Hoque et al. (2020) found that *Acinetobacter* was the predominantly abundant genus in the milk of Bangladesh cows with clinical mastitis. In this study, spearman’s correlation analyses showed that *Acinetobacter* was positively correlated with the human_pathogens_all function, and negatively correlated with nitrogen_fixation, reductive_acetogenesis functions. This indicates that EA supplementation may promote intestinal health in lactating mares by reducing *Acinetobacter* abundance and increasing intestinal acetogenesis. Christensenellaceae, a potential biomarker of gut health, is highly heritable and plays a vital role in maintaining gut health (Chen et al., 2024). *Christensenellaceae_R_7_group* belongs to the Christensenellaceae family and is one of the five taxa that characterize the healthy gut. Because it contains genes for essential cellulase and hemicellulase secreting enzymes that can break down fibrous material, ferment fiber and produce SCFAs, which have multiple beneficial effects on animal performance and gut health (Evans et al., 2011; Liu et al., 2021; van der Hee and Wells, 2021). In this study, spearman’s correlation analysis showed that the *Christensenellaceae_R_7_group* was positively correlated with serum PROG and PRL levels, GSH-Px activity, and milk PRL levels. Coriobacteriales_Incertae_Sedis has strong bidirectional interactions with polyphenols and is associated with the production of SCFAs and bile acid metabolism (Rodríguez-Daza et al., 2020; Miao et al., 2022; Zhao et al., 2024). Although the biological function of *Phoenicibacter* remains unclear, in this study found that *Phoenicibacter* was positively correlated with serum and milk of PRL levels, as well as with fecal contents of acetate, propionate, isobutyrate, butyrate, isovalerate, valerate, and TVFA. Additionally, it was negatively correlated with milk MDA levels. These correlations indicate that *Christensenellaceae_R_7_group* and *Phoenicibacter* may influence lactation-related hormone regulation and antioxidant capacity in lactating mares through SCFAs-mediated pathways. Studies have shown that SCFAs can directly or indirectly modulate neural and endocrine functions via the microbiota-gut-brain (MGB) axis (Dalile et al., 2019), thereby potentially regulating the secretion of hormones such as PRL. Furthermore, SCFAs-particularly butyrate-can enhance the expression of antioxidant enzymes (including the glutathione peroxidase (GPX) family), by activating signaling pathways such as GPR109A/AMPK/Nrf2 (Guo et al., 2020; Shaw and Chattopadhyay, 2020). Although the direct causal relationship remains to be elucidated and verified by further research, these findings suggest a potential interaction mechanism between gut microbes and their metabolites (SCFAs) and host physiological responses during lactation. LEfSe analysis in this study revealed that Bacillales predominated in the CON lactating mares, while Christensenellaceae was enriched in EA-supplemented mares, consistent with the above results. Tax4Fun functional predictions showed that microbial functions in EA-supplemented lactating mares were primarily associated with fiber and carbohydrate degradation. Microbial degradation of fiber and carbohydrates is the main pathway for the production of SCFAs (Akhtar et al., 2022). This aligns with the observed increases in acetate, propionate, isobutyrate, butyrate, isovalerate, valerate, and TVFA in the feces of these mares.

## 5 Conclusion

In conclusion, EA supplementation in lactating mares improves milk production, regulates lactation-related hormone secretion, and enhances antioxidant capacity. Additionally, EA alters gut microbiota composition of lactating mares, stimulates SCFA production, inhibits harmful bacteria proliferation, and promotes intestinal health. These effects ultimately improve the production performance and health of lactating mares. Under the conditions of this study, a supplementation dose of 15 mg/kg BW EA is optimal for lactating mares, suggesting that EA may serve as an effective feed additive for lactating mare production. However, it should be acknowledged that this study has certain limitations: the relatively small sample size and the limited duration of the experiment may restrict the generalizability of the findings and the assessment of the long-term effects of EA supplementation. Additionally, as the study was conducted with a specific breed and under particular feeding conditions, the applicability of the results may be limited.

Regarding the practical applications of these findings, although pure EA showed potential as a feed additive in this study, its economic feasibility in large-scale equine production may be limited by production costs. Therefore, exploring EA-rich plant byproducts (such as pomegranate peel, walnut peel, chestnut peel, and berry pomaces from strawberry, cranberry, and blackberry) as alternative sources could be a more cost-effective strategy. Future research should compare the efficacy and economic benefits of pure EA versus plant byproducts. Overall, EA supplementation holds promise as a nutritional intervention to improve lactation performance and health in lactating mares. Further studies addressing the limitations mentioned above should involve larger cohorts, extended feeding periods, and cost-benefit analyses to validate the efficacy and practicality of EA supplementation in equine husbandry.

## Data Availability

The raw sequencing data has been submitted into the National Center for Biotechnology Information sequence read archive under PRJNA1123219.

## Glossary

CAT: Catalase
CON: The control group receiving no EA supplementation
E2: Estradiol
EA: Ellagic acid
EA 15 group: Each mare was supplemented with EA 15 mg/kg BW every day
EA 30 group: Each mare was supplemented with EA 30 mg/kg BW every day
GH: Growth hormone
GSH-Px: Glutathione peroxidase
LDA: Linear discriminant analysis
LEfSe: Linear discriminant analysis coupled with effect size
MDA: Malondialdehyde
NMDS: Nonmetric multidimensional scaling
OTUs: Operational taxonomic units
PCA: Principal component analysis
PCoA: Principal coordinate analysis
PRL: Prolactin
PROG: Progesterone
SCFAs: Short-chain fatty acids
SOD: Superoxide dismutase
T-AOC: Total antioxidant capacity
TVFA: Total volatile fatty acids
